# Impact of High-Resolution Epiaortic Ultrasonographic Imaging on Evaluating Aortic Wall Pathology

**DOI:** 10.3400/avd.cr.21-00112

**Published:** 2022-03-25

**Authors:** Soichiro Henmi, So Izumi, Ryoichi Mizoue, Yutaka Okita, Kenji Okada, Takuro Tsukube

**Affiliations:** 1 Division of Cardiovascular Surgery, Japanese Red Cross Kobe Hospital, Kobe, Hyogo, Japan; 2 Department of Anesthesiology, Japanese Red Cross Kobe Hospital, Kobe, Hyogo, Japan; 3 Division of Cardiovascular Surgery, Takatsuki General Hospital, Takatsuki, Osaka, Japan; 4 Division of Cardiovascular Surgery, Kobe University Graduate School of Medicine, Kobe, Hyogo, Japan

**Keywords:** aortic dissection, tunica media, high-resolution epiaortic ultrasonography

## Abstract

There has been no definitive method, other than pathological findings, to identify the degeneration of the tunica media in the aortic wall (TM). We describe how high-resolution intraoperative epiaortic ultrasonographic imaging identifies changes in the TM of patients with aortic dissection. This method shows great promise in facilitating presymptomatic diagnoses of various aortic wall pathologies.

## Introduction

We previously reported that synchrotron radiation-based X-ray phase-contrast tomography (XPCT) imaging revealed densitometrical changes in the tunica media in the aortic wall (TM) of patients with acute type-A aortic dissection (AADA).^1,2)^ However, the application of XPCT entails the use of hazardous levels of radiation. To replicate XPCT findings in clinical practice, we investigated the application of high-resolution ultrasound in intraoperative epiaortic ultrasonographic (EAU) imaging.

## Case Report

Three patients (A: normal aorta, B: chronic aortic dissection, C: AADA) were selected. Before beginning cardiopulmonary bypass, the midascending aorta was exposed and an EAU examination was conducted using EPIQ CVx and an eL18-4 transducer (maximum frequency: 22 MHz, theoretical axial resolution 35 µm, Philips Ultrasound, Inc., Bothell, WA, USA). Further image processing and analysis of the ultrasonographic intensity (UI) data were performed using ImageJ software (http://rsbweb.nih.gov/ij/index.html).

**Figure 1:**
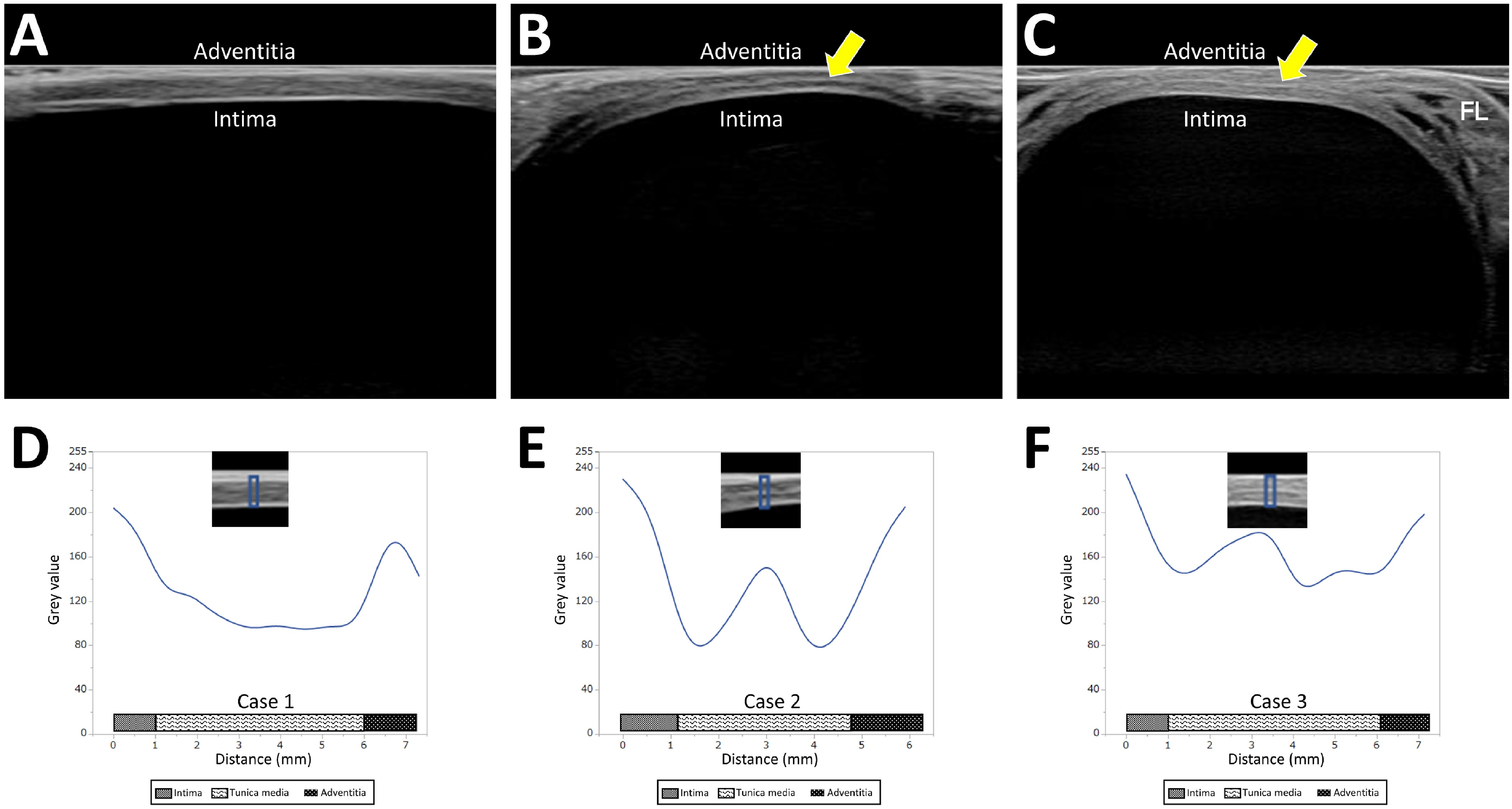
Fig. 1 High-resolution epiaortic ultrasound imaging and line profile of ultrasound intensity of three patients. (**A**) Case-1 with no aortic pathology. (**B**) Case-2 with chronic aortic dissection. High-intensity linear changes were observed in the middle of the tunica media (indicated by the yellow arrow). (**C**) Case-3 with acute type-A aortic dissection. The tunica media was separated according to the line of high intensity (yellow arrow). (**D**) A line profile of the intensity from the intima to the adventitia (indicated by the blue square) in Case-1. (**E**) A line profile of the intensity (blue square) in Case-2. (**F**) A line profile of the intensity (blue square) in Case-3.

In Case-1, a 51-year-old male with aortic and mitral valve regurgitation underwent a double valve replacement. EAU imaging showed that the UI remained unchanged throughout the TM (**Fig. 1A**). In Case-2, a 47-year-old female with chronic dissection in the ascending aorta underwent a valve-sparing aortic root replacement. EAU imaging of the aorta section that had not been dissected clearly showed high linear changes in the UI in the middle of the TM (**Fig. 1B**). In Case-3, a 71-year-old female with AADA underwent a root and hemiarch replacement. High linear changes in the UI were observed in the middle of the TM, and aortic dissection was observed as an extension of those linear changes (**Fig. 1C**). In summary, EAU imaging of the aortic wall in patients with AADA clearly shows high linear changes in the UI in TM, even at the nondissected aortic wall; compared with these findings, that of the aortic wall in patients without AADA does not show it. A line profile of the UI between the intima and adventitia showed that the UI remained unchanged in Case-1 (**Fig. 1D**) and exhibited a high peak in the middle of TM in Case-2 (**Fig. 1E**) and Case-3 (**Fig. 1F**). These results indicated clear differences in the UI throughout TM between normal and pathological aortas.

FL: false lumen of the aorta

## Discussion

Intraoperative EAU imaging of the ascending aorta is a widely applied strategy for reducing atherosclerotic emboli.^3)^ However, it has not been used to evaluate the TM of the aortic wall. This study is the first to use recently available high-resolution ultrasound in EAU imaging to visualize changes in the UI throughout TM. These findings were consistent with changes in TM of patients with AADA, which were obtained using XPCT.^2)^ Therefore, high-resolution ultrasonographic imaging could surrogate XPCT findings in clinical settings and lead to significant improvements in the presymptomatic diagnosis of various aortic wall pathologies, including aortic dissection.

## Conclusion

High-resolution intraoperative epiaortic ultrasonographic imaging could show great promise in facilitating presymptomatic diagnoses of various aortic wall pathologies.

## Additional Note

We have obtained written informed consent from these patients for reporting the data.
